# Aerobic condition enhances bacteriostatic effects of silver nanoparticles in aquatic environment: an antimicrobial study on *Pseudomonas aeruginosa*

**DOI:** 10.1038/s41598-017-07989-w

**Published:** 2017-08-07

**Authors:** Zhaoyu Chen, Ping Yang, Zhiguo Yuan, Jianhua Guo

**Affiliations:** 1 Department of Environmental Science & Engineering, Sichuan University, Chengdu, Sichuan 610065 China; 2 Advanced Water Management Centre, The University of Queensland, St Lucia, Brisbane QLD 4072 Australia

## Abstract

The intensive applications of silver nanoparticles (AgNPs) inevitably cause continuous release of such materials into environments, as a consequence posing potential risks to microbial communities in engineered or natural ecosystems. However, the magnitude of antibacterial capacity of nanoparticles is still inconclusive, owing to influential factors such as the size of nanoparticle, microbial species, or environmental conditions. To reveal whether the presence of air would alter AgNPs ecotoxicity, *Pseudomonas aeruginosa* PAO1, a facultative denitrifying bacterium and an opportunity pathogen, was used to study antibacterial assays under both anaerobic and aerobic conditions. The results indicate that the respiration status of *P. aeruginosa* affect the ecotoxicity of AgNPs. *P. aeruginosa* cultured under aerobic condition were more susceptible to AgNPs than that under anaerobic condition. Aerobic condition greatly enhanced bacteriostatic effects of AgNPs but not their bactericidal effects, as the ratio of viable but nonculturable (VBNC) bacteria remained above 90% when 5 mg L^−1^ AgNPs applied. Our findings offer further understanding for the degree of toxicity of nanoparticles on microbial ecosystems and underscore the importance of exposure condition (e.g. oxygen) in the mode of action of AgNPs.

## Introduction

Silver nanoparticles (AgNPs) have been widely used in consumer products such as textile, cosmetics, and medical products, due to their excellent antibacterial, optical, and electronic properties^1–3^. AgNPs are the most popular nanoparticles in markets, which accounts for about 50% of commercial nanoproducts^4^. It is estimated that approximate 500 tons of AgNPs were produced each year worldwide^5, 6^. As being incorporated in fabric and personal care products, the processes of fabrication and consumption thereby can release AgNPs from consumer products to sewage, eventually entering environment. Thus, it represents a potential risk to a great variety of organisms in natural or engineered ecosystems, including wastewater treatment plants (WWTPs).

AgNPs have shown great antibacterial activities against various microbes. Choi *et al*.^7^ reported that the respiration of autotrophic nitrifiers in a continuously stirred tank reactor was inhibited by 86% at 1 mg L^−1^ of AgNPs. Tan *et al*.^8^ documented that AgNPs at 300 *µ*g L^−1^ decreased nitrifying efficiency from 98% to 15%. In addition, Li *et al*.^9^ showed that a commercial AgNPs at 10 mg L^−1^ was the miminum inhibitory concentration (MIC) to *Escherichia coli*, whereas a synthesized AgNPs exhibited 100% killing of *E. coli* when added at 4.5 mg L^−1^ ^10^. According to these previous studies^7–11^, the concentration of AgNPs that will cause toxic impacts on microbes, ranged from *µ*g L^−1^ to mg L^−1^. Such an inconclusive antibacterial capacity of nanoparticles may be associated with different exposure conditions or microorganism types, which may alter the antibacterial activity of nanoparticles. Some studies^12–15^ suggested that the susceptibility of microbes, microbial adaptability, the synthesis and coating of NPs, and dissolution of metal cations in aqueous solution environment would affect the ecotoxicity of AgNPs. For instance, Lu *et al*.^15^ investigated the antibacterial effects of AgNPs by exposure of five oral pathogenic bacteria to AgNPs, and corresponding MICs varied from 25 to 50 mg L^−1^. Arnaout and Gunsch^16^ tested impacts of AgNP coatings (gum arabic, polyvinylpyrrolidone, and citrate) on *Nitrosomonas europaea*, and found that citrate AgNPs had a stronger inhibition on nitrification and caused more serious membrane disruption, compared to AgNPs coated with gum Arabic or polyvinylpyrrolidone.

Although the ecotoxicity of AgNPs has attracted more and more attention, it is so far still not clear if different respiratory conditions (aerobic vs anaerobic) can affect the toxicity of AgNPs. Zero-valent AgNP is insoluble in water, but Ag^+^ can be chemisorbed, or liberated from AgNPs into liquid especially in the presence of oxygen^17^. Thus, the aerobic and anaerobic conditions may interfere with the effects of AgNPs on microbial activities through changing the dissolved Ag^+^ in their living environment^18^. There seems a general agreement in previous studies^19, 20^ that the dissolved Ag^+^ is the factor leading to the toxicity of AgNPs. However, both anaerobic and aerobic conditions exist in natural or engineered ecosystems, thus microbial communities would be easily exposed to these two conditions. As most bacteria in WWTPs are facultative bacteria, it is of great importance to investigate the effects of anaerobic and aerobic conditions on the difference of antibacterial effects of AgNPs to facultative microorganisms, considering previous studies reported differential degree of toxicity.

The objective of this study is to examine the response of environmental bacteria to AgNPs, and to compare the toxicity of AgNPs under anaerobic versus aerobic conditions. Pure culture investigation can give us insights into the potential mechanisms of AgNPs antibacterial action toward microbes. Therefore, *Pseudomonas aeruginosa* PAO1 (*P. aeruginosa*), a ubiquitous denitrifier in WWTPs contributing to nitrogen removal and also an opportunistic pathogen often found in hospital environment^21^, was used as a model bacterium to evaluate how exposure condition affects the action of AgNPs. Their dose-response patterns are compared under anaerobic and aerobic conditions based on antibacterial assays using physiological and metabolic approaches.

## Results

### Cell growth profile and cell viability

**Figure 1:**
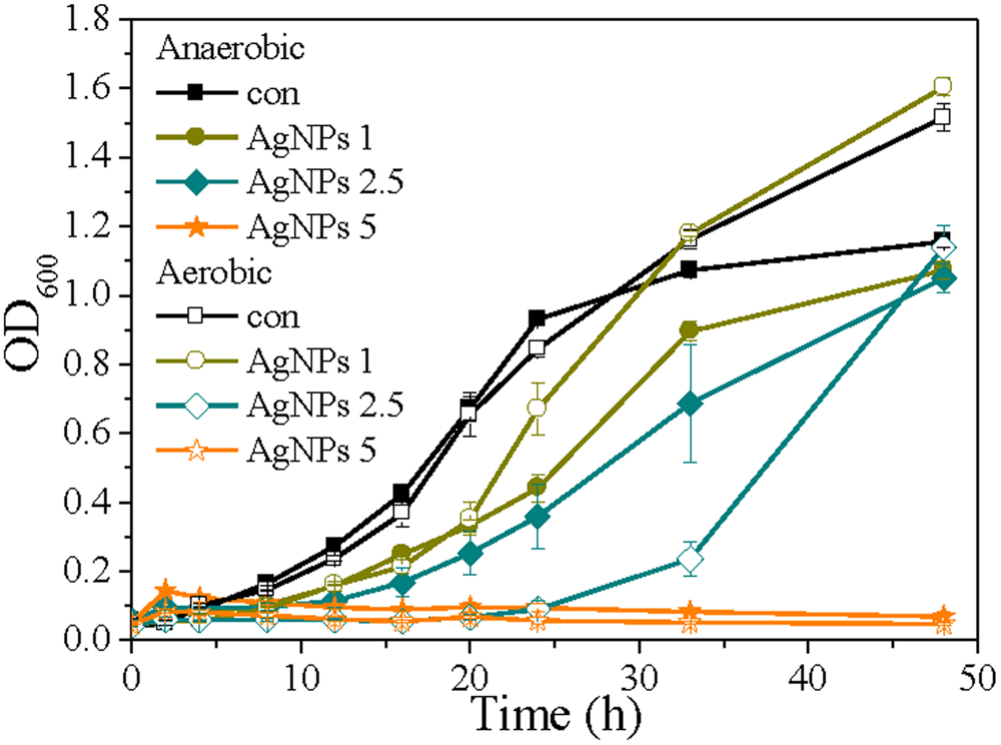
OD_600_ profiles in *P. aeruginisa* cultures with different levels of AgNPs (mg L^-1^) under anaerobic versus aerobic conditions. Data are shown as mean values ± standard deviations that are deduced from three independent experiments.

To investigate antibacterial property of AgNPs under anaerobic versus aerobic conditions, OD_600_ (optical density at 600 nm) profiles were monitored to refelect the status of cell growth (Fig. 1). In the absence of AgNPs, *P. aeruginosa* cultured aerobically obtained a higher growth plateau compared to anaerobic control after 48 h cultivation, indicating *P. aeruginosa* has a relatively quicker growth rate under aerobic condition. Adding 1 mg L^−1^ AgNPs into cultures extended the lag phase and consequently postponed the beginning of exponential growth, despite of air condition. Compared to anaerobic condition, cells under aerobic condition still grew in a faster rate. However, when AgNPs amendments were increased to 2.5 mg L^−1^, aerobically cultured *P. aeruginosa* apparently suffered more inhibition, in comparison to that of cells under anaerobic condition. For example, after 24 h cultivation, the OD_600_ value of bacteria under aerobic condition was notably lower than that under anaerobic condition (*p* = 0.03). Furthermore, the growth of *P. aeruginosa* was totally inhibited in the presence of 5 mg L^−1^ AgNPs, irrespective of the presence or absence of oxygen.

**Figure 2:**
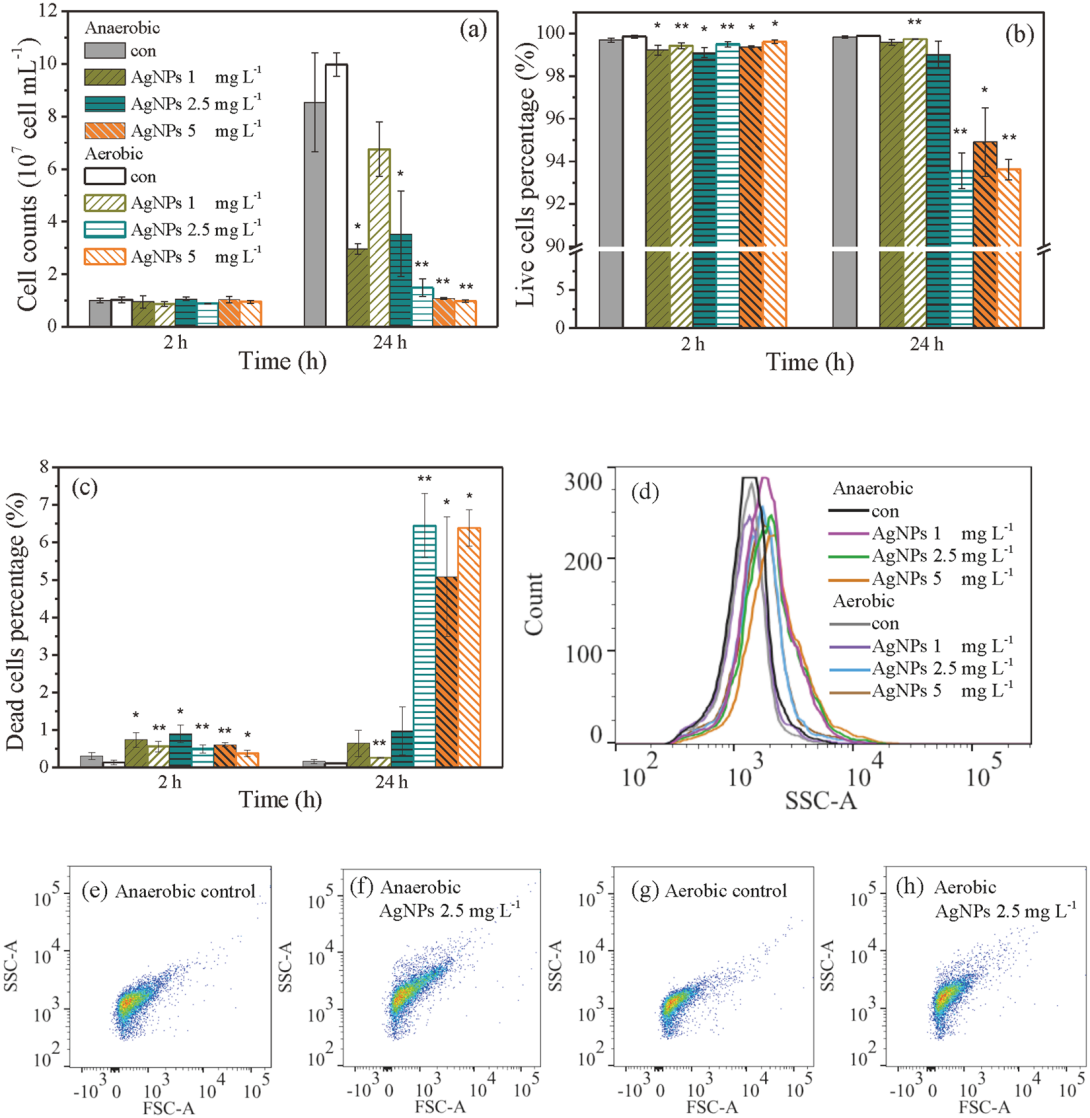
Total cell number (**a**), live cell ratio (**b**), and dead cell ratio (**c**) in *P. aeruginisa* cultures with different levels of AgNPs under anaerobic versus aerobic conditions. Side scatter statistics analysis (**d**), and FSC and SSC analysis (**e**–**h**) of cells cultivated 2 h in cultures with different levels of AgNPs under anaerobic versus aerobic conditions. Data are shown as mean values ± standard deviation, which are deduced from three independent experiments. Asterisks indicate significant differences compared to control samples (one-way ANOVA, *p < 0.05, **p < 0.01). The legend in (**a**) also applies to (**b**) and (**c**).

The results based on flow cytometry (Fig. 2) further refelected the effects of AgNPs on the growth of *P. aeruginosa*, which were consistent with OD profiles (Fig. 1). For controls without AgNPs, *P. aeruginosa* grew much faster under aerobic condition than under anaerobic condition. As shown in Fig. 2a, the total cell counts for all cultures showed no significant difference upon 2 h exposure of AgNPs. However, after 24 h cultivation, the dosage of AgNPs under anaerobic and aerobic conditions caused different growth rates. Slightly higher cell density (10.0 × 10^7^ cells mL^−1^) and cell growth rate (4.1 × 10^6^ cells mL^−1^ h^−1^) were observed in aerobic controls compared to that (8.5 × 10^7^ cells mL^−1^ and 3.4 × 10^6^ cells mL^−1^ h^−1^, respectively) in anaerobic controls (*p* > 0.05). *P. aeruginosa* cultures amended with AgNPs at a concentration of 1 mg L^−1^ also obtained a faster growth rate under aerobic respiration, albeit this dosage level ocuured lag phase extension. However, this trend reversed with the addition of AgNPs of 2.5 mg L^−1^, which caused a stronger inhibition on cell proliferation under aerobic condition. It is noted that with the increasing addition of AgNPs from 1 to 2.5 mg L^−1^, bacteria cultured anearobically exhibited slight increase in cell growth rate (from 9.1 × 10^5^ to 11.2 × 10^5^ cells mL^−1^ h^−1^) whereas those cells cultivated aerobically declined significantly (from 2.7 × 10^6^ to 2.6 × 10^5^ cells mL^−1^ h^−1^). It implies that 1 and 2.5 mg L^−1^ of AgNPs have similar inhibitory effects on cell growth under anaerobic condition. However, aerobic condition exerts an increased stress on cell growth with the AgNPs concentrations. Consistent with OD profiles, flow cytometry resutls showed that AgNPs at 5 mg L^−1^ completely hindered cell growth under both conditions as the total cell number remained at the same level during 24 h exposure.

In order to further understand the physiological status of cells in various cultures, LIVE/DEAD staining was performed to observe cell viability (Fig. 2b and c). High live cell ratios (>99%) were detected in all cultures after 2 h exposure, independent of anaerobic or aerobic conditions. After 24 h growth, the live cell ratio maintained at the same level in controls and cultures amended with 1 mg L^−1^ AgNPs. Under exposure of 2.5 mg L^−1^ AgNPs, the live cell ratio was still high (99%) in anaerobically cultivated cells, wheras that of the aerobically cultivated cells encountered a decline (93%). With the AgNPs concentration being increased to 5 mg L^−1^, the live cell ratio descreased to 95% under anaerobic condition, while that kept at 93% under aerobic condition. The dead cell ratios of treated cultures were shown in Fig. 2c. Collectively, the flow cytometry results indicate that PAO1 grown aerobically suffered more repression when exposed to AgNPs at 2.5 mg L^−1^; while AgNPs at 5 mg L^−1^ caused shut down of cell multiplication of PAO1 but did not affect cell viability to the same extent, indicating cells were in viable but nonculturable (VBNC) status.

During the FACS (fluorescent-activated cell sorting) detection process, cells were examined by FSC (forward scatter) and SSC (side scatter) detectors and represented in SSC distribution (Fig. 2d) and FSC vs. SSC density plots (Fig. 2e–h). According to SSC distribution, increments in SSC were observed in cells exposed to AgNPs, which may due to light reflected from AgNPs internalized by the bacteria^22, 23^. The FSC vs. SSC density plots of cells in controls and cultures amended with AgNPs (2.5 mg L^−1^), under anaerobic or aerobic conditions, were demonstrated in Fig. 2e–h. It was observed that the FSC signal, indicating relative dieffernces in cell size, was no significant change in AgNPs experiment. However, the SSC signal slightly increased when AgNPs added, irrespective of the presence or absence of oxygen.

### Impacts of AgNPs on metabolism of *P. aeruginosa*

**Figure 3:**
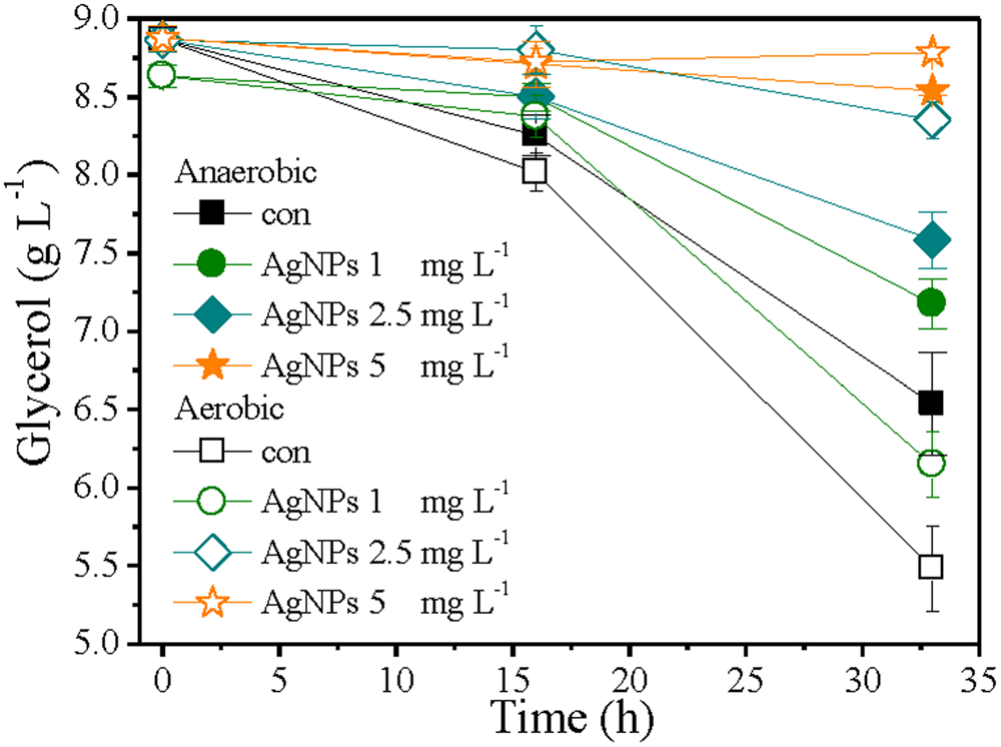
Variations of glycerol concentration in *P. aeruginosa* cultures in the presence of different levels of AgNPs under anaerobic versus aerobic conditions.

To assess how the AgNPs influence bacterial metabolism, carbon source consumption was monitored during the exposure period. Consistent with the cell growth profiles, the glycerol consumption (Fig. 3) decreased with elevated NPs dosage. The glycerol consumption of aerobic control was 36.9 mM L^−1^ after 33 h cultivation, compared to 25.4 mM L^−1^ in anaerobic control (*p* > 0.05). Although the dosage of 1 mg L^−1^ AgNPs extended the lag phase of *P. aeruginosa* growth under aerobic condition, its glycerol consumption (27.0 mM L^−1^) exceeded anaerobic control (25.4 mM L^−1^) after 33 h cultivation, consitent with OD_600_ values (Fig. 1). However, aerobic cultures amended with 2.5 mg L^−1^ AgNPs consumed 5.6 mM L^−1^ glycerol after 33 h exposure, which represented only 40% of that consumed by the anaerobic cultures exposed 2.5 mg L^−1^ AgNPs (13.9 mM L^−1^). AgNPs at 5 mg L^−1^ totally hindered cell growth under both conditions, with very negligible carbon source consumption.

### TEM imaging of control and AgNPs treated *P. aeruginosa*

**Figure 4:**
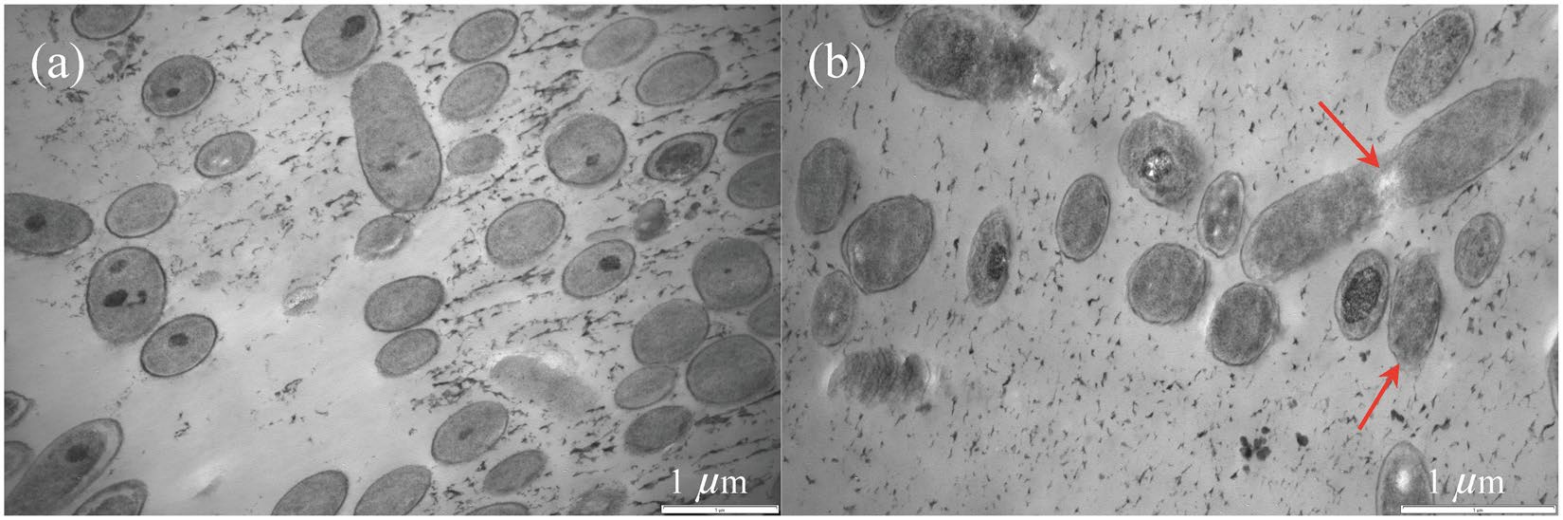
Representative transmission electronic microscopy images of *P. aeruginosa* under 6 h exposure in the absence (**a**) and presence (**b**) of AgNPs (2.5 mg L^-1^). Arrows point to cells with damage membrane or lysed.

To understand alterations in cell morphology or structure after AgNPs treatment under anaerobic and aerobic conditions, TEM images (Fig. 4) of control and treated cells were recorded. It is observed that the oxygen presence did not make distinct difference to cell morphology based on TEM images, in which cells having similar structural properties under anaerobic and aerobic conditions. However, the variations between cells in controls and suspensions amended with AgNPs (2.5 mg L^−1^) were noticeable. The addition of AgNPs resulted in membrane damage of *P. aeruginosa* cells (Fig. 4). Control cells (Fig. 4a) have a single morphology with predominantly spherical shapes and undamaged membranes. However, *P. aeruginosa* exposed to 2.5 mg L^−1^ AgNPs displayed some signs of membrane disruption and rough membrane surface (as shown in Fig. 4b). In addition, a few numbers of cells appeared to have lysed potentially with leaks of intracellular materials. Therefore, the addition of AgNPs posed detrimental effects on morphological properties of *P. aeruginosa* cells, maybe leading to the inhibition of cell growth in AgNPs (2.5 mg L^−1^) treated cultures.

### Discerning the toxicity of leaching Ag^+^ under aerobic versus anaerobic conditions

Discerning the contribution of AgNPs versus leaching Ag^+^ to biological effects is challenging due to their common co-occurrence^24^. To reveal potential involvement of silver ions liberated from AgNPs on cell growth inhibition, the levels of Ag^+^ released in the media were determined (Table 1). For all cases, the leaching of Ag^+^ increased with prolonged exposure time. However, the amount of released Ag^+^ did not show distinct increases with the AgNP concentrations either under aerobic or anaerobic condition (*p* > 0.05). Almost all exposure conditions obtained similar amount of Ag^+^ release (i.e. 0.110 ~ 0.124 mg L^−1^) after 24 h exposure (Table 1).

**Table 1:** Comparisons of leaching Ag^+^ (mg L^-1^) under aerobic versus anaerobic conditions based on ICP-MS approach.

	Aerobic-1 mg L^-1^	Aerobic-2.5 mg L^−1^	Aerobic-5 mg L^−1^	Anaerobic-1 mg L^−1^	Anaerobic-2.5 mg L^−1^	Anaerobic-5 mg L^−1^
2 h	0.096 ± 0.005	0.079 ± 0.006	0.112 ± 0.023	0.064 ± 0.007	0.078 ± 0.007	0.060 ± 0.011
24 h	0.110 ± 0.005	0.124 ± 0.009	0.120 ± 0.010	0.080 ± 0.005	0.118 ± 0.010	0.115 ± 0.010

## Discussion

Since the antibacterial property of silver nanoparticles reported in literature was contradictory, it is of great significance to understand factors that will influence NPs performance. In order to explore potential effects of air on AgNPs’ ecotoxicity to bacterial viability and metabolic properties relevant to wastewater biological treatment, we exposed *P. aeruginosa*, a model bacterial organism, to AgNPs ranged from 1 to 5 mg L^−1^ under aerobic or anaerobic conditions. The results reflected aerobic condition enhanced the bacteriostatic activity of AgNPs. In addition, AgNPs at 2.5 mg L^−1^ seemed to be the threshold that AgNPs began to exert a strong inhibition to aerobic cell growth and cell activities. Xiu *et al*.^18^ also reported that aerobic condition increased toxicity of AgNPs on *E. coli* compared to anaerobic condition. They hypothsised that anaerobic condition precludes Ag^+^ release, which was believed to contribute to AgNPs stress.

It is also observed that AgNPs inhibited bacterial activities under anaerobic condition, indicating the general toxicity of AgNPs. The repression shown in bacterial growth (Figs 1 and 2) and metabolism (Fig. 3) in cultures amended with AgNPs imply that, although selected circumstances (aerobic or anaerobic) will differentially affect the degree of ecotoxicity, AgNPs (5 mg L^−1^) also have strong ecotoxicity under anaerobic condition. Lu *et al*.^15^ also reported that AgNPs exhibited some antibacterial activity against five anaerobic oral pathogenic bacteria by dosing 25 ~ 50 mg L^−1^ as the minimum inhibition concentration (MIC). In contrast, some studies suggested that AgNPs generally would not affect the performance of anaerobic biological treatment systems^25^, since an anaerobic bioreactor may prevent silver ion release^18^ or transform AgNPs into silver sulfide^26^, which may provide detoxification mechanisms for AgNPs.

Although 93% of the total cells were still viable in suspensions amended with AgNPs of 5 mg L^−1^, cell growth was eventually not displayed, implying that the increasing AgNPs dosage did not correspond to an increase in cell death ratio but accelerated the transition to VBNC status. Similar results were found when performing high concentrations (2 mg mL^−1^) of TiO_2_-NPs to *Pseudomonas putida*, that nearly all the cell population were viable but only one third of them were cultivable^27^. It has been previously reported that the VBNC state plays an critical role in the survival of bacteria, and in their ability to affect environment and human health^28, 29^. In VBNC cells, the transport and biosynthesis system, and the ability to utilize substrates are maintained. It is usually accompanied by a reduction in metabolic activity levels to minimize cellular energetic requirements^30^, which is an survival strategy of many bacteria in response to adverse environmental conditions. Thus, the resuscitation of VBNC cells would allow them regain cultuability and, as *P. aeruginosa* a pathogen, renewed ability to induce infection^28^. Further studies on these VBNC cells resulted from the stress of AgNPs would be interesting.

The detection of Ag^+^ leaching levels from AgNPs was performed to determine whether Ag^+^ associated with the differential displayed toxicity of NPs under anaerobic versus aerobic conditions. Table 1 indicates that Ag^+^ was not the key determinant that caused the different ecotoxicity between two culturing conditions. The anaerobic condition was supposed to eliminate confounding effects associated with oxidative release of silver ions^13^. However, except oxidative dissolution of AgNPs caused silver ion, the chemisorbed silver ions from the surface of AgNPs is another Ag^+^ release mechanism^17^. The synthesis approaches may also affect the silver ions distribution in the media. In the present study, released Ag^+^ was detectable under anaerobic conditions at the beginning of exposure (2 h) even at lower dosage level (1 mg L^−1^), and the amount of leaching Ag^+^ reached to the same level with that under aerobic condition after 24 h. Specifically, AgNPs at 2.5 mg L^−1^ released comparable amount of Ag^+^ under aerobic and anaerobic condition at 2 or 24 h exposure. However, this dosage induced a stronger inhibition on aerobic cell growth in terms of OD and live cell numbers, compared with anaerobic condition. In addition, under anaerobic condition, similar levels (0.115 ~ 0.118 mg L^−1^) of Ag^+^ were detected when exposed to AgNPs of 2.5 and 5 mg L^−1^ for 24 h, while 5 mg L^−1^ of AgNPs appeared to be more toxic to *P. aeruginosa*. These results indicate that anaerobic condition could not eliminate Ag^+^ release from AgNPs as expected, and the released Ag^+^ was not the dominant bacteriostatic mechanism of AgNPs, which is different with the previous report suggesting that Ag^+^ released from AgNPs was mainly responsible for the toxicity of AgNPs^18^.

The mechanism for the enhanced antibacterial activity of AgNPs under aerobic condition is still unclear. One possible explanation is based on the negative influence of reactive oxygen species (ROS) on cellular constitutes and cell membrane^11, 31^. Oxygen condition may increase the intracellular ROS production in cells^4^. In this study, ROS detection assay was conducted, estimated by 2′,7′-dichlorofluorescindiacetate (H_2_-DCFDA, ab113851, Abcam), to check variations of cellular ROS. However, treated cells had no distinct difference in ROS level compared to the controls (data not shown). So it could rule out ROS induction as a possible mechanism for acute toxicity of AgNPs to *P. aeruginosa*. Yang *et al*.^20^ also did not detect ROS induction when exposed *Pseudomonas stutzeri, Azotobacter vinelandii*, and *Nitrosomonas europaea* to AgNPs with 0.5 mg L^−1^. Another proposed explanation is the abrasive surface texture of AgNPs^14^, which has more uneven and rough surface texture when exposed to air^24^. This surface roughness may relate to the mechanical damage of cell membrane as shown in TEM images (Fig. 4), the cell membrane integrity of *P. aeruginosa* treated with 2.5 mg L^−1^ AgNPs was less intact compared with cells in the controls. Given the anaerobic and aerobic exposure can influence the magnitude of AgNPs’ ecotoxicity to microorganisms, the manipulation of air condition present potential role in adjusting antibacterial efficacy of AgNPs. The widespread application of AgNPs makes increasing quantities of nano-materials released into WWTPs, thus, the management of oxygen condition could be beneficial for controlling the growth of facultative bacteria in these regions or for reducing the risk of NPs caused ecotoxicity.

In summary, this study documented that aerobic condition enhances bacteriostatic property of silver nanoparticles, compared to anaerobic condition. AgNPs at 2.5 mg L^−1^ exhibited an enhanced ecotoxicity to *P. aeruginosa* under aerobic condition, suffering inhibition on cell proliferation and lower cell viability in terms of flow cytometry results. Correspondingly, carbon source utilization encountered more suppression under aerobic condition. In addition, AgNPs also exert antibacterial influences when bacteria grow anaerobically. Physiological changes in *P. aeruginosa* were observed with the transition of cells to VBNC status when AgNPs posed inhibition on bacteria. Results obtained from ICP-MS detection revealed that silver ion has a negligible contribution to the detrimental performance of AgNPs in this study. The findings offer improved understanding on the magnitude of AgNPs toxicity on microbial ecosystems and underscore the importance of aqueous solution environment (e.g. oxygen) in the mode of AgNPs antibacterial activity.

## Materials and Methods

### Nanoparticles

Silver nanoparticles dispersion (10.16%, w/w) was obtained from Fraunhofer, Germany. The AgNPs were synthesized by an aqueous reduction method. To make AgNPs stock dispersion of 1 mg mL^−1^, purchased dispersion was diluted in Milli-Q water and homogenized by ultrasonication (80 W) for 1 h in water bath (20 °C). These particles have a mean size of 40 nm in the stock dispersion, determined by dynamic light scattering with a Zetasizer Nano ZS (Malvern, UK).

### Bacterial strain and culturing condition

*P. aeruginosa* strain (DSM No: 22644), as the model microorganism for AgNPs experiments, was obtained from the Deutsche Sammlung von Mikroorganismen und Zellkulturen GmbH, Germany. Following the supplier’s instructions, the stain was activated by growing on tryptic soy agar (TSA) plates at 30 °C for 26 h. A single colony on TSA plates was inoculated to tryptic soy broth (TSB) and grown in a shaking incubator (30 °C, 150 rpm) overnight. Afterwards, bacterial suspension (5 mL) in TSB was transferred into serum bottles fed with 150 mL of sterile anaerobic glycerol modified M9 (GLYM9) media^32^ in an anaerobic chamber and incubated overnight. The optical density at 600 nm (OD_600_) of this culture was then adjusted to 0.5 and multiple 10 mL aliquots were transferred respectively to each serum bottle and cell culture flask (with vent cap, Corning^®^) containing 150 mL sterile GLYM9 medium. All cultures were incubated at 30 °C.

The ingredients of GLYM9 were 10 g L^−1^ glycerol, 30 mM NaNO_3_, 0.2 g L^−1^ yeast extract, 0.2 M phosphate buffer, 9 mM NaCl, 38 mM NH_4_Cl, 2 mM MgSO_4_·3H_2_O, and 100 *µ*M CaCl_2_·2H_2_O^32^. To prepare aerobic media, GLYM9 were autoclaved and then using the biosafety cabinet to transfer media into sterile cell culture flasks with vent cap (Corning), which has 0.2 *µ*m pore hydrophobic membrane allowing air exchange meanwhile minimizing contamination. For anaerobic conditions, GLYM9 media were decanted into serum bottles and followed by sparging with nitrogen gas for 30 min before autoclave sterilization.

### *P. aeruginosa* treated with silver nanoparticles

Sterile culture media in serum bottles (anaerobic) and flasks with vent cap (aerobic) were inoculated, in anaerobic chamber or biosafety cabinet respectively, with bacterial suspension from overnight pre-cultures to achieve an initial cellular concentration of about 10^7^ cells mL^−1^. To observe the interactions between AgNPs and bacteria, various volumes of AgNPs stock dispersion were injected in 150 mL GLYM9 media, immediately after the inoculation of bacteria, to obtain different starting concentrations of 1, 2.5 and 5 mg L^−1^. These concentrations of AgNPs were chosen on the basis of preliminary studies (data not shown), expressing the spectrum of cell growth responses to AgNPs. Control cultures had no amendments. *P. aeruginosa* cultures were further incubated for 48 h under either anaerobic or aerobic conditions. All experiments were performed in triplicate. During the exposure period, samples were collected from triplicate cultures at nine different time points (sampling interval of 2 or 4 h) for determination of cell growth and viability, and supernatants were used for determination of glycerol and silver ions levels, as described below.

### Physiological assays

OD_600_, as an indicator of cell density, was measured during the exposure period by using a Cary 50 bio UV visible spectrophotometer (Varian, Australia). The final concentration of AgNPs in the media resulted in an acceptable scattering error contribution (<10%) for the OD_600_ absorbance (<0.01). Cell density and viability of *P. aeruginosa*, non-amended and amended with AgNPs, were measured using LIVE/DEAD staining (BacLight^TM^ Bacterial Viability Kit, L7012). In brief, triplicate cultured cells were stained with 1.5 *µ*L each of SYTO 9 (S9) and propidium iodide (PI) per 1,000 *µ*L sample, and incubated in the dark for 15 min at room temperature before the measurement by a FACSAria^TM^ II flow cytometer (BD Biosciences, San Jose, USA). The determinations of cell concentrations in suspensions were performed by adding fluorescent microspheres (100 beads per 1 *µ*L). Thus, the cell counts of *D. vulgaris* can be calculated by the ratio of bacterial events to microsphere events. To determine Live/Dead ratio of cells, cells located in specific gate were expressed as a proportion of total cells, as described in the previous study^21^. FACSAria^TM^ II flow cytometer (BD Biosciences, San Jose, USA) contains 5 lasers, two of which (Blue 488 nm and Yellow/Green 561 nm) were used in this study, (forward scatter) FSC detector and (side scatter) SSC detector. As each cell in samples untercepts the laser beam, laser light is scattered in all derections. The FSC parameter was used as an indicator of cell size, whereas SSC parameter was used to indicate cell complexity^27^.

### Metabolic assays

During the cultivation period, the concentration of glycerol was monitored to reflect the metabolism of *P. aeruginosa*. The suspensions of *P. aeruginosa* cultures were immediately filtered through 0.22 *µ*m filters (Merck Millipore, USA). Glycerol concentrations were detected by a high-performance liquid chromatography (HPLC), which is a Shimadzu LC equipped with an Agilent Hi-Plex H column (300 × 7.8 mm) specialized for organic acid analysis.

### Transmission electron microscopy (TEM) imaging of *P. aeruginosa*

To compare the different morphology of *P. aeruginosa* cells amened with AgNPs under aerobic versus anaerobic conditions, suspension samples were collected at 6 h cultivation and directly centrifuged at 5,000 rpm for 5 min. Samples for TEM imaging were performed by standard procedures, and details are similar with the previous study^33, 34^. Briefly, after discharging supernatants, pellets were resuspended by agar and deposited on a copper grid, immediately frozen via a high-pressure freezer and then freeze-dried. After embedded in epoxy resin, ultrathin sections (50 ~ 100 nm) of samples were then cut and dropped off on TEM copper grid and imaging was performed by TEM using a JEOL JEM-1011 (JEOL, Tokyo, Japan) operated at 80 kV.

### ICP-MS

Inductively coupled plasma mass spectrometry (ICP-MS) (Agilent 7700) was performed to quantify silver ions leaching from AgNPs under both aerobic and anaerobic conditions. Aliquots of media amended with AgNPs under different culture conditions were centrifuged (20,000 rpm, 20 min). Supernatants were then filtered through centrifugal filter (3 kDa, Amicon, Germany) to capture nanoparticles^26^. Filtrates were brought to containers with 1 mL of concentrated nitric acid to acidify and digest metal cations to measure total dissolved silver ions.

### Statistical analysis

Results are presented as mean (± standard deviation) from triplicate experiments. Statistical significance was determined by one-way analysis of variance (ANOVA) for the total cell counts and viability.

### Data availability

The datasets generated during and/or analysed during the current study are available from the corresponding author on reasonable request.
